# Editorial: Signaling in roots: the brain of the plant

**DOI:** 10.3389/fpls.2026.1952143

**Published:** 2026-08-18

**Authors:** Madhusmita Panigrahy, Kishore C. S. Panigrahi, Antonio Montagnoli

**Affiliations:** 1 Institute of Agricultural Science, Siksha O Anusandhan deemed to be University, Bhubaneswar, Odisha, India; 2 School of Biological Sciences, National Institute of Science Education and Research, Jatni, Odisha, India; 3 Laboratory of Environmental and Applied Botany, Department of Biotechnology and Life Science, University of Insubria, Varese, Italy

**Keywords:** abiotic stress adaptation, climate-resilient crops, nutrient homeostasis, plant–microbe interactions, rhizosphere interactions, root phenotyping and plasticity, root signaling, root system architecture

Roots are crucial to plant productivity, maintaining nutrient balance through adaptive changes in root system architecture (RSA) (Bagale et al., 2025; Beatrice et al., 2024; Dumroese et al., 2019). As agriculture strives to ensure food security while minimizing environmental impacts, understanding and harnessing root traits have become vital for improving nutrient efficiency, optimizing biogeochemical cycles, and remediating polluted sites, including those affected by eutrophication (Amendola et al., 2017; Baesso et al., 2018, 2020; Ceriani et al., 2025; Dalle Fratte et al., 2022; Griffiths et al., 2022).

Over recent decades, root research has expanded beyond classical studies of root architecture and nutrient acquisition to include gene regulatory networks (Cassan et al., 2023), hormonomics (De Zio et al., 2019; De Nittis et al., 2025; Hirayama and Mochida, 2023), and enzymatic regulation. Together, these disciplines advance understanding of root development and cell patterning, root-rhizosphere and root-microbe interactions (Gillini et al., 2025; Mourouzidou et al., 2025; Sferra et al., 2025), and the mechanisms underlying root regeneration and repair (Efroni et al., 2016; García-Gómez and ten Tusscher, 2024; Zhi and Hu, 2023). A rapidly evolving area of research examines how developmental and environmental signals are integrated to regulate root growth and function (Bricca et al., 2023; Ma et al., 2024; Agusti et al., 2021; Montagnoli et al., 2019, 2023; Nyam-Osor et al., 2021). Recent studies have further highlighted the importance of epigenetic mechanisms in environmental adaptation, nutrient signaling, and responses to self-DNA (Qadir et al., 2026; Kajrolkar, 2025). In addition, epigenetic memory and heritable epialleles are providing new insights into the long-term adaptive capacity of the root system under changing environmental conditions (Hemenway and Gehring, 2023; Vaschetto, 2024).

Advances in rhizometric analysis, including digital imaging and computational approaches, have greatly improved the characterization and quantification of root growth (Gao et al., 2025; Paez-Garcia et al., 2015). These techniques allow the measurement of root diameter, branching patterns, growth angles, topological depth, and overall architecture (Montagnoli et al., 2024). These methods enable systematic qualitative root analysis and provide dynamic information on 2D root growth and turnover (Terzaghi et al., 2025).

Root architecture is increasingly recognized as a key determinant of nutrient homeostasis and plant adaptation to environmental stress. In particular, deep-rooted RSA contributes to plant resilience under drought and other adverse climatic conditions (Zhang et al., 2025). High-throughput phenotyping approaches are therefore becoming valuable tools for crop improvement (Galkovskyi et al., 2012). Li et al. developed a high-throughput paper-pouch-based phenotyping platform to assess drought tolerance in 28 wheat genotypes. Their results demonstrated the effectiveness of this approach in distinguishing tolerant and sensitive genotypes based on multiple deep-root architectural traits. Drought-tolerant genotypes exhibited a distinctive cortical configuration identified through a multi-trait evaluation framework. Early-stage screening using such phenotyping platforms could facilitate the identification of promising targets and support correlations with field performance across breeding programs. Root-neighbor interactions are widely thought to be mediated by root exudates, which shape belowground ecological dynamics and influence root growth and allelopathic responses (Ma et al., 2022). Giorgi et al. investigated metabolic shifts in hydrophilic and lipophilic root exudates during allelopathic interactions between *Sorghum bicolor* and the invasive weed *Cyperus rotundus*. The authors developed a protocol to isolate root exudates from solid growth media and showed that root proximity induced specific metabolic changes rather than a general increase in exudation. Under the tested conditions, these interactions did not cause immediate growth inhibition or significant biomass reductions in either species. This pioneering non-targeted metabolic profiling study using 1H-NMR spectroscopy provides a valuable framework for investigating root-neighbor interactions and their implications for plant productivity in agroecosystems. Zhang et al. examined how increasing nitrogen application affects nutrient partitioning and root structural characteristics in rubber plantations. Nitrogen treatments at high, medium, and low levels were evaluated across different developmental stages and plant organs, including roots, stems, and bark, as well as during dormancy. Their findings challenge the conventional reliance on high nitrogen inputs, revealing that medium N application improved plant growth, latex production, root architecture, nitrogen storage dynamics, and resilience to environmental stress while reducing fertilizer requirements. Future validation under field conditions and across multiple growing seasons will be necessary to confirm the potential of these practices for sustainable and climate-smart rubber production. Plant growth-promoting rhizobacteria (PGPR) are increasingly recognized for their role in enhancing plant performance and stress tolerance (Das et al., 2024). Wang et al. demonstrated that inoculation with a PGPR consortium alleviated salt stress in rice. They proposed a PGPR-rice metabolic mutualism model in which PGPR-induced root exudation recruited beneficial microbiota taxa, including *Subgroup_7*, *Lysobacter*, *Massilia*, thereby enhancing plant growth and development. Recruitment of a stress-protective microbiome, optimized nutrient acquisition, effective reactive oxygen species (ROS) scavenging, and the potential establishment of synthetic microbial communities were identified as key mechanisms underlying saline soil reclamation. These findings further support the potential application of PGPR-based strategies in crop production under stressful environmental conditions. Cadmium-contaminated soils represent a major threat to food safety and plant productivity. Heavy-metal accumulator species have therefore been extensively investigated for their phytoremediation potential (Das et al., 2022). Ren et al. demonstrated that microalgae application in hydroponic systems enhanced phytoremediation efficiency, Cd accumulation, and plant growth in *Perilla frutescens*. These improvements were associated with increased antioxidant activity, altered root exudation patterns, and enhanced secretion of defense-related metabolites. Further studies under field conditions are needed to evaluate the scalability and applicability of this approach across different crop species and contaminated environments. Zheng et al. reviewed developments in hairy root research from 2009 to 2024 through a bibliometric analysis of Web of Science publications. By examining contributions according to countries, institutions, journals, authors, and keywords, they identified major trends, emerging research frontiers, and future collaboration opportunities. Owing to their high transformation efficiency, gene plasticity, and growing relevance in CRISPR-Cas9 gene-editing applications, hairy roots represent a valuable platform for functional genomics and secondary metabolite production. The study also highlighted challenges that remain to be addressed, including species-dependent gene-editing efficiencies and metabolite stability during large-scale cultivation. Because drought remains a major constraint on crop productivity under climate change scenarios (Masante et al., 2026; Wang et al., 2024), McCubbin et al. employed a divided-container system to investigate nodal root growth in *Zea mays* under water-deficit conditions. Their results showed that, unlike primary roots, nodal root elongation is maintained through sustained meristematic cell production and increased net solute deposition, facilitating osmotic adjustment and continued tissue expansion. This response was associated with transcriptional and metabolic changes related to osmolyte accumulation, hormone signaling, and ROS homeostasis. Metabolomic analysis further revealed taurine accumulation and proline biosynthesis via the saccharopine pathway, indicating that acclimation mechanisms predominantly support nodal root growth under drought stress. Inorganic phosphorus (Pi), another essential yet frequently limiting nutrient, also drives adaptive modification in RSA (Upadhyay et al., 2025; Panigrahy et al., 2009). Previous studies have shown that light exposure can aggravate Pi deficiency responses by promoting accumulations of Fe, callose, lignin, and hydroxyl radicals in roots (Gao et al., 2021). In this context, Wang et al. demonstrated that inhibition of primary root growth under Pi-deficiency conditions is primarily driven by light exposure rather than by shoot-derived signals, whereas blue-light signaling itself contributes only marginally. Transcriptomic analyses suggested that blue light-induced chemical reactions underlie the observed root growth inhibition. These findings call for a possible re-evaluation of experimental methodologies involving illuminating Petri dishes for the study of root nutrient responses. The AP2/ERF family comprises a large group of plant-specific transcriptional regulators that integrate developmental and environmental signals into specific gene-expression regulations. Members of this family play central roles in growth, development, and stress responses, including ethylene-mediated signaling. Fan et al. showed that the *slerf4–9* mutant exhibited impaired root architecture and reduced seedling growth, accompanied by decreased expression of the auxin transport genes SlAEC2 and SlPIN5 and altered auxin distribution. Their findings suggest that SlERF4–9 regulates root development indirectly through the modulation of auxin transport, providing new insights into the transcriptional control of root growth. Finally, Endo et al. advanced our understanding of iron homeostasis, a critical process for plant growth and survival (Przybyla-Toscano et al., 2021). Investigating MEB3, a member of the vacuolar iron transporter (VIT) family in *Arabidopsis thaliana*, the authors found that iron concentrations, but not zinc levels, were reduced in the roots of *meb3* knockout mutants, indicating impaired iron storage capacity. Their results further suggest that MEB3 may contribute to the long-distance transport of iron from roots to shoots, highlighting its important role in maintaining iron homeostasis.

Collectively, the contributions in this Research Topic highlight significant advances in root biology, spanning fundamental mechanisms, methodological innovation, and practical applications. By integrating insights from phenotyping, molecular biology, physiology, and plant–environment interactions, these studies reinforce the central role of roots in nutrient acquisition, stress adaptation, rhizosphere dynamics, and ecosystem functioning. Beyond expanding our understanding of root development and signaling, the findings underscore the potential of root-focused research to support sustainable agriculture, environmental remediation, and the development of climate-resilient cropping systems. The growing integration of functional genomics, microbiome research, metabolomics, and advanced phenotyping is creating new opportunities to link root traits to plant performance across diverse environments. Despite this progress, important challenges remain. A deeper understanding of how roots integrate developmental and environmental signals, interact with the rhizosphere, and maintain functional plasticity is still needed. Equally important is translating discoveries from controlled experiments to field-scale applications. Addressing these challenges will require stronger integration across molecular, physiological, ecological, and technological disciplines. As environmental pressures intensify, closing these knowledge gaps will be essential to developing innovative strategies that enhance plant resilience, resource-use efficiency, and ecosystem sustainability.
